# Systematic Review and Meta-Analysis of the Effectiveness of Calcium-Phosphate Coating on the Osseointegration of Titanium Implants

**DOI:** 10.3390/ma14113015

**Published:** 2021-06-02

**Authors:** Nansi López-Valverde, Antonio López-Valverde, Juan Manuel Aragoneses, Bruno Macedo de Sousa, María João Rodrigues, Juan Manuel Ramírez

**Affiliations:** 1 Department of Surgery, Instituto de Investigación Biomédica de Salamanca (IBSAL), University of Salamanca, 37007 Salamanca, Spain; nlovalher@usal.es; 2 Faculty of Dentistry, Universidad Alfonso X El Sabio, 28691 Madrid, Spain; jmaragoneses@gmail.com; 3 Polo I-Edifício Central Rua Larga, Institute for Occlusion and Orofacial Pain Faculty of Medicine, University of Coimbra, 3004-504 Coimbra, Portugal; brunomsousa@usal.es (B.M.d.S.); maria.jrodrigues@hotmail.com (M.J.R.); 4 Department of Morphological Sciences, University of Cordoba, Avenida Menéndez Pidal S/N, 14071 Cordoba, Spain; jmramirez@uco.es

**Keywords:** titanium dental implant, calcium-phosphate coating, osseointegration

## Abstract

Ca-P coatings on Ti implants have demonstrated good osseointegration capability due to their similarity to bone mineral matter. Three databases (PubMed, Embase, and Web of Science) were searched electronically in February 2021 for preclinical studies in unmodified experimental animals, with at least four weeks of follow-up, measuring bone-to-implant contact (BIC). Although 107 studies were found in the initial search, only eight experimental preclinical studies were included. Adverse events were selected by two independent investigators. The risk of bias assessment of the selected studies was evaluated using the Cochrane Collaboration Tool. Finally, a meta-analysis of the results found no statistical significance between implants coated with Ca-P and implants with etched conventional surfaces (difference of means, random effects: 5.40; 99% CI: −5.85, 16.65). With the limitations of the present review, Ca-P-coated Ti surfaces have similar osseointegration performance to conventional etched surfaces. Future well-designed studies with large samples are required to confirm our findings.

## 1. Introduction

Titanium (Ti) is one of the most widely used materials for the manufacture of dental and orthopaedic implants due to its mechanical properties, chemical stability, and excellent biocompatibility [1]. The quality of implants depends on the properties of their surfaces; therefore, the modification of these surfaces, with the aim of achieving optimal osseointegration and shortening waiting times for functional loading, has become an area of great interest for researchers and is under constant evolution.

The osseointegration of implants has been defined as a direct and functional connection between the bone and the implant, where the macroscopic and microscopic characteristics of the implant surface are of great importance. Lack of osseointegration is often due to poor bone formation around the implant surface, leading to insufficient fixation of the implant [2].

The deposition of calcium-phosphate (Ca-P) coatings on the implant surface has received significant attention due to the chemical similarity to natural bone mineral. Ca-P-based coatings show the ability to adhere directly to bone tissue and to increase the biochemical anchorage between bone and the coating material [3]. Ca-P coatings on titanium implants have been shown to improve their biofunctionality by facilitating osseointegration and longevity, hence the existing philosophy regarding this type of coating is that biological integration is improved when the structure mimics bone [4,5,6].

Ca-P, in the form of apatite, is the main mineral content (~69%) of natural bone [7]. However, it is not osteoinductive [8], and its activity is limited to osteoconduction, although it has been shown that, in combination with growth factors and bioactive proteins, it can be osteoinductive [9].

Ultrastructural observations have shown that Ca-P coatings partially dissolve, saturating body fluids in the peri-implant area and leading to a double precipitation of biological apatite, which could serve as a substrate for bone-forming cells, the only difficulty being matching the dissolution of the coating with the rate of healing to achieve ideal bone apposition on the titanium surface [10].

Although previous reviews on this topic have been published, none of them compared in vivo, Ca-P-coated Ti surfaces with conventional etched surfaces (sandblasted large grit acid etched, SLA, surfaces). Therefore, the aim of the present systematic review and meta-analysis was to bring together preclinical studies in experimental animals to determine whether Ca-P-coated Ti implant surfaces possess increased osseointegration capability.

## 2. Materials and Methods

This systematic review and meta-analysis were conducted in accordance with the Preferred Reporting Items for Systematic Reviews and Meta-Analyses (PRISMA) guidelines [11].

### 2.1. Protocol and Registration

A search was carried out for any registered protocols on a similar topic in the International Prospective Register of Systematic Reviews (PROSPERO). No systematic review protocols were found in this database. Therefore, this review was pre-registered in the PROSPERO platform under the identification number CRD-REGISTER-2-ID255185.

The Population, Intervention, Comparison, Outcome and Study Design framework (PICOS) was used as a basis to formulate the research question, which was: “Do Ca-P-coated Ti surfaces have a higher osseointegration capacity than etched Ti surfaces?”. (P) Population: animals receiving endosseous Ti implants. (I) Intervention: Ti implants with Ca-P incorporation. (C) Comparison: Ti implants with conventional surface. (O) Outcome: bone formation around the implant surface. (S) Study design: preclinical studies in unmodified experimental animals (Table 1).

### 2.2. Inclusion and Exclusion Criteria

The inclusion criteria for the study selection were:

- Preclinical studies in unmodified animals (osteoporotic, diabetic…), using endosseous implants with Ca-P incorporation;
- Studies with at least six animals and 4 weeks of follow-up;
- Studies published in English.

The exclusion criteria for the study selection were:

- In vitro studies;
- Narrative and systematic reviews;
- Clinical cases;
- Studies that did not meet the established inclusion criteria.

### 2.3. Search Strategy

The following search strategy was used: Two independent researchers conducted electronic searches in the PubMed, Embase, and Web of Science (WoS) databases up to February 2021 with the Medical Subject Headings (MeSH) terms: “Titanium implants”, “biocompatible coated materials”, “osseointegration”, “calcium phosphate”, “animal model”. Boolean operators “AND” and “OR” were used to refine the search (Table A1).

### 2.4. Selection of Studies

Two independent reviewers (N.L.-V., A.L.-V.) carried out the study selection by obtaining full text data from the selected articles, including general information, animal parameters (total number, species), methods of Ca-P incorporation, timing of assessment, methods of analysis, conclusions, and implant parameters (total number, length, diameter, shape, location, and surface characteristics of implant and control). After eliminating duplicates, studies were selected according to the inclusion criteria. Cohen’s kappa statistic was calculated to measure the level of agreement between the two reviewers. Disagreement on the eligibility of studies was resolved by discussion between the two reviewers.

### 2.5. Risk of Bias

The Systematic Review Centre for Laboratory Animal Experimentation (SYRCLE) risk of bias tool, an adapted version of the Cochrane RoB tool with specific biases in animal studies), was used to assess the methodology of the scientific evidence in all selected studies [12].

### 2.6. Quality of the Reports of the Selected Articles

Animal Research: Reporting of In Vivo Experiments (ARRIVE) [13] guidelines were used, with a total of 23 items. Each item was scored by reviewers N.L.-V. and A.L.-V. with scores of 0 (not reported) or 1 (reported), with an overall inventory of all included studies (Table 1).

### 2.7. Statistical Analysis

Odds ratios (ORs) with 95% confidence intervals (CI) were used for adverse event outcomes. The mean difference (MD) and standard deviation (SD) for BIC were used to estimate effect size. The meta-analysis was performed using RevMan software (Review Manager version 5.3; The Cochrane Collaboration, Copenhagen, Denmark). The random-effects model was selected because of the expected methodological heterogeneity in the included studies; furthermore, significant heterogeneity was interpreted when the I^2^ value was > 50% [14]. The threshold for statistical significance was defined as *p* < 0.05. A funnel plot was used to assess publication bias.

## 3. Results

### 3.1. Selection and Description of Studies

**Figure 1:**
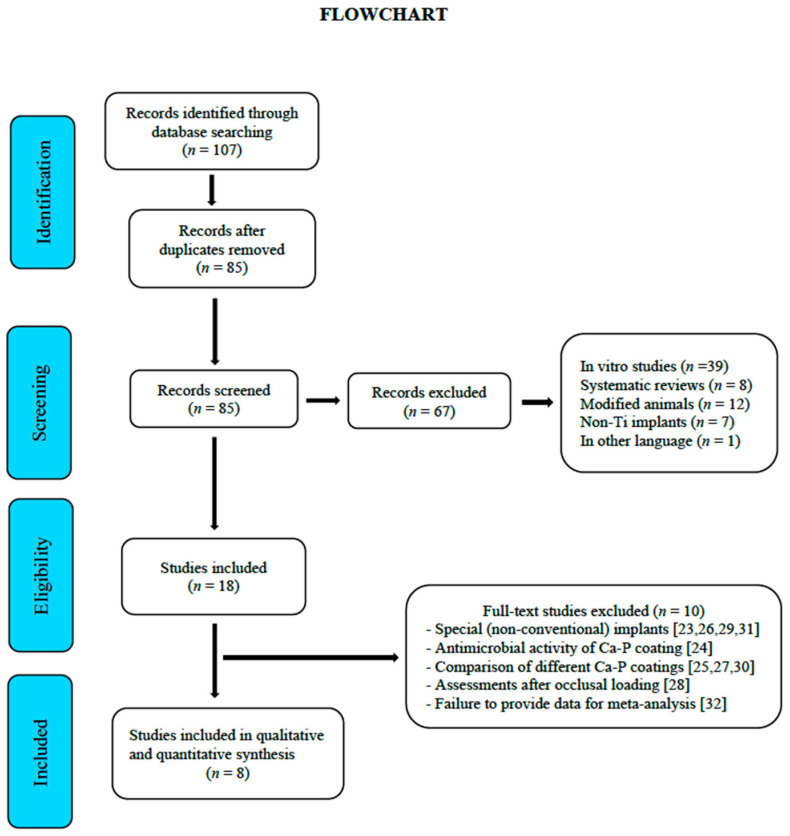
Flowchart.

The initial electronic search yielded 107 references. After eliminating duplicates and irrelevant articles based on their title and abstracts (in vitro studies, systematic reviews, modified animals, non-Ti implants, and articles in other languages), 18 full texts were selected [15,16,17,18,19,20,21,22,23,24,25,26,27,28,29,30,31,32]. The concordance between reviewers (N.L.-V., A.L.-V.) was 100% with a Cohen’s kappa index of 1 (overall concordance). The reasons for rejecting 10 studies out of the 18 selected were the following: use of unconventional implants [23,26,29,31], comparing different apatite veneers [25,27,30], assessing parameters after occlusal loading [28], assessing the antimicrobial activity of the Ca-P veneer [24], and not providing data for meta-analysis [32]. Finally, eight studies were selected for the meta-analysis [15,16,17,18,19,20,21,22] (Figure 1).

Table 2 provides the assessment of the ARRIVE criteria in animal studies, with a mean score of 17.25 ± 0.46. All studies provided adequate information in terms of title, abstract, introduction, ethical statement, species, surgical procedure, outcome assessment, and statistical analysis. Items 5 (rationale for animal models), 19 (3Rs, replace, reduce and refine), 20 (adverse events), 21 (limitations of the study) and 22 (generalizability/applicability) were not reported in any of the included studies.

### 3.2. Risk of Bias Assessment

**Figure 2:**
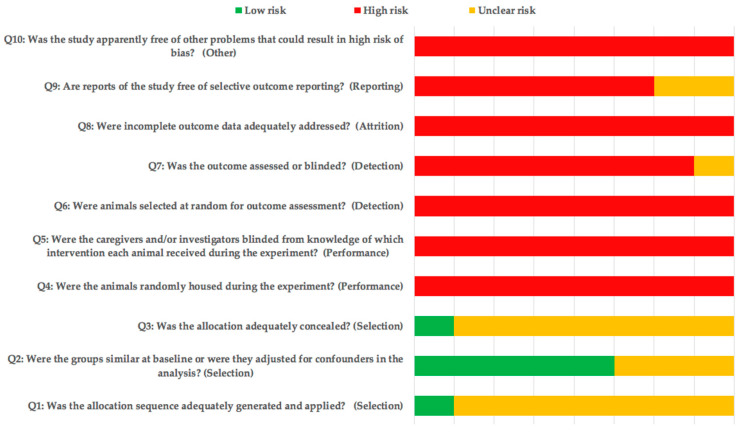
SYRCLE’s risk of bias tool.

Although item 2 was mentioned in several studies, the lack of information resulted in a high and unclear risk of bias for most of the included studies (Figure 2).

### 3.3. Qualitative Synthesis

The most commonly used animal model was rabbit [15,16,17,20], and all included studies evaluated BIC (Table 3); two of the included studies [17,21] evaluated bone density (BD) and two bone area (BA) [15,18]. All implants used were commercial threaded implants and only one of the studies used hydroxyapatite (HA) in combination with calcium oxide (CaO) as a coating [15]. The methods of Ca-P incorporation to the surface of the experimental implants were different in all selected studies (Table 4).

### 3.4. Quantitative Synthesis (Meta-Analysis)

**Figure 3:**
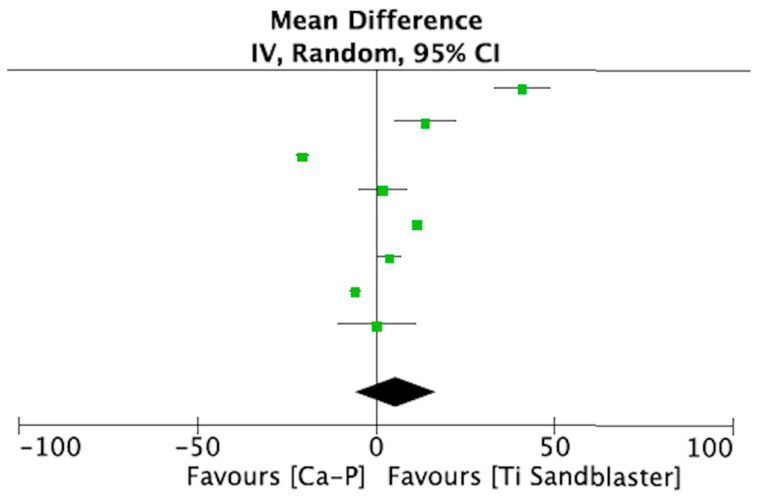
Forest plot for meta-analysis of studies evaluating BIC at 4 weeks after placement, assuming a random-effects model.

The same studies included in the qualitative synthesis were used to perform a meta-analysis comparing Ca-P-coated Ti implants with etched Ti implants, with a total of 455 implants being evaluated. A meta-analysis of adverse outcomes could not be performed due to lack of data. All included studies [15,16,17,18,19,20,21,22] assessed BIC 4 weeks after placement. Heterogeneity was very high (I^2^ = 99%) (Table 5)). Figure 3 shows the forest plot for the meta-analysis.

### 3.5. Publication Bias and Heterogeneity

**Figure 4:**
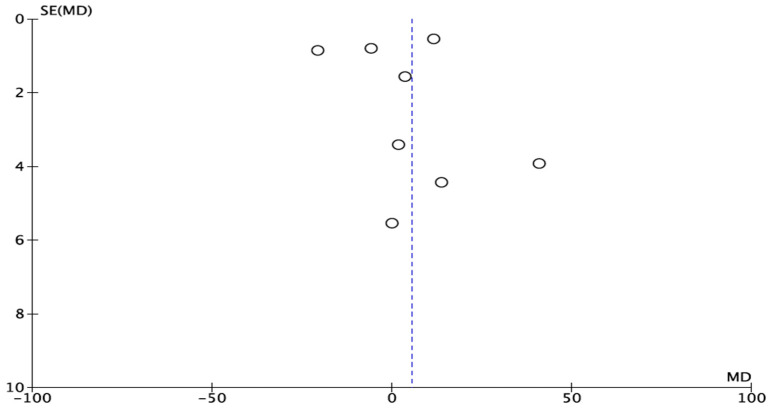
Funnel plot for BIC. The asymmetry proves publication bias.

The experimental studies show graphical signs of publication bias, as can be observed in the funnel plot (Figure 4).

## 4. Discussion

The purpose of the present study was to answer the following clinical question: “Do Ca-P-coated Ti surfaces have a higher osseointegration capacity than etched Ti surfaces?”. To quantify the potential effect of Ca-P-containing surfaces on peri-implant bone apposition, a meta-analysis of BIC was performed. Our meta-analysis found no statistical significance between implants coated with Ca-P and implants with conventional etched surfaces (SLA type).

Certain thin Ca-P coatings have been shown to be amorphous and readily soluble in simulated body fluids [33], and several studies have found no difference in early osseointegration between CA-P-coated implants and implants with etched or Ti powder-blasted surfaces [34,35,36]. Koh and colleagues [15] in a study in rabbits concurred with these findings, finding no difference in bone apposition around Ca-P-coated surfaces compared to etched surfaces. Various forms of Ca-P differ in solubility and stability, which are characteristics that alter their biocompatibility. HA is a very poorly soluble but very stable Ca orthophosphate. Schliephake and colleagues [21] in a study in dogs compared the BIC of Ca-P and HA-coated implants and uncoated implants, finding no significant differences between the groups.

However, many studies have shown that Ca-P coatings improve the biocompatibility and fixation of implants; for example, Vercaigne and colleagues reported that Ca-P coatings are much more effective in stimulating the bone reaction than microroughness of surfaces [37], emphasizing that, in addition to the implant surface conditions, the bone reaction to an oral implant is determined by the local conditions at the implantation site, i.e., the presence of cortical or trabecular bone [38]. However, not all types of coatings achieve the same results. The coating technique is another important factor that can alter the solubility and stability of the coating [39]. Micro-coatings appear to improve fixation in the first few weeks by increasing the bone-to-implant contact surface [40], although these types of coatings tend to crack easily, detaching the coating and leading to implant failure [41,42]. Despite this, each technique has different advantages and disadvantages in terms of processing and outcome. However, coarse-grained coatings are the most prone to fracture of the bone-coating-metal substrate interface long after implantation, which has led to this type of implant falling into disuse in clinical practice. A study by Coelho and colleagues [43] compared the biological response of Ti alloy (Ti-6Al-4V) cylinders with Ca-P deposition cylinders in a dog model, determining the BIC using a computer program, and found no significant differences between the two surfaces compared in the first weeks of implantation.

Research seeks to improve the biomechanics of bone tissue by designing implants with improved biocompatibility, osteoconductivity and osteoinductivity, leading to faster and improved bone healing and turnover [44,45].

After implantation, the implant surfaces come into contact with biological fluids and tissues, and there are two types of host response: either forming an intermediate fibrous layer that does not guarantee adequate biomechanical fixation or direct bone-to-implant contact, ensuring osseointegration [4]. However, the actual process of osseointegration remains an unknown and little-studied mechanism, with genetics being identified as one of the inherent variables in the patient [46].

Numerous studies have shown that early fixation and long-term mechanical stability of fixtures are improved with rough profiles compared to smooth surfaces [47,48]; however, rough surfaces are more prone to generating pathologies around the implant tissues (peri-implantitis), and this would work against Ca-P-coated surfaces [49].

After implantation of Ca-P-coated fixtures, a layer of biological apatite is released on the implant surface that could serve as a matrix for adhesion and growth of osteogenic cells [50].

The studies included in our meta-analysis used different coating processes: oxidation [15,16], micro-coating (particle deposition) [18,19,20,21], and blasting (plasma spray) [20]. The study by Poulos and colleagues [17] used a proprietary coating method not described in the study; however, it has been described that the plasma spray technique is not very effective for coating dental implants with complex topographies [51], making it very difficult at this time to present a detailed discussion on the commercialization of Ca-P coatings, films, and layers on commercial Ti implants [52]. However, mechanical conditions are not the only requirement for promoting bone response. Implants with a thin Ca-P coating resulted in the highest amount of bone contact, but it is difficult to give a definitive explanation for the coating effect of Ca-P ceramics [36].

Finally, it should be added that we encountered some serious limitations related to this meta-analysis: firstly, the small number of studies included and therefore the limited number of implants studied; second, the high risk of bias of all studies; third, the substantial heterogeneity of the selected studies. This did not allow any solid conclusions to be drawn.

## 5. Conclusions

Within the aforementioned limitations, it can be concluded that Ca-P-coated Ti surfaces have a similar osseointegration power to conventional etched surfaces (SLA or similar). However, in order to confirm our results, well-structured, well-conducted studies with larger samples and longer follow-ups are necessary.

## Figures and Tables

**Table 1:** PICOS items.

Population (P)	Unmodified animals (osteoporotic, diabetic…) receiving endosseous titanium implants.
Intervention (I)	Ti implants with Ca-P incorporation.
Comparison (C)	Ti implants with conventional etched surfaces (SLA type).
Outcomes (O)	Bone formation around the implant surface (bone-to-implant contact, BIC).
Study design (S)	Preclinical studies with at least six animals and 4 weeks follow-up.

**Table 2:** Checklist of ARRIVE criteria reported by the included studies. Each item was judged as “0” (not reported) or “1” (reported). The total score of each of included studies was also recorded.

Studies	Koh et al. 2013 [15]	Fontana et al. 2011 [16]	Poulos et al. 2011 [17]	Quaranta et al. 2010 [18]	Fügl et al. 2009 [19]	Le Guehennec et al. 2008 [20]	Schliephake et al. 2006 [21]	Caulier et al. 1997 [22]
1. Title	1	1	1	1	1	1	1	1
Abstract								
2. Species	1	1	1	1	1	1	1	1
3. Key finding	1	1	1	1	1	1	1	1
Introduction								
4. Background	1	1	1	1	1	1	1	1
5. Reasons for animal models	0	0	0	0	0	0	0	0
6. Objectives	1	1	1	1	1	1	1	1
Methods								
7. Ethical statement	1	1	1	1	1	1	1	1
8. Study design	1	1	1	1	1	1	1	1
9. Experimental procedures	1	1	1	1	1	1	1	1
10. Experimental animals	1	1	1	1	1	1	1	1
11. Accommodation and handling of animals	0	1	1	0	1	0	0	0
12. Sample size	1	1	1	1	1	1	1	1
13. Assignment of animals to experimental groups	1	1	1	1	1	1	1	1
14. Anesthesia	1	1	1	1	1	1	1	1
15. Statistical methods	1	1	1	1	1	1	1	1
Results								
16. Experimental results	1	1	1	1	1	1	1	1
17. Results and estimation	1	1	1	1	1	1	1	1
Discussion								
18. Interpretation and scientific implications	1	1	1	1	1	1	1	1
19. 3Rs reported	0	0	0	0	0	0	0	0
20. Adverse events	0	0	0	0	0	0	0	0
21. Study limitations	0	0	0	0	0	0	0	0
22. Generalization/applicability	0	0	0	0	0	0	1	0
23. Funding	1	0	1	1	0	1	1	1
Total score	17	17	18	17	17	17	18	17

Mode value: 17.25 ± 0.46.

**Table 3:** Characteristics of the studies included.

Studies	Animal Model	Implants (*n*)	Follow-Up (Weeks)	Analysis Methods	Conclusions
Koh et al. 2013 [15]	Rabbit model (6)	12	2 and 4	Histomorphometry BIC	A Ca-P coating on an anodized surface may induce rapid osseointegration at the bone-implant interface and more bone formation near the implant surface.
Fontana et al. 2011 [16]	Rabbit model (36)	216	2, 4, and 9	Histomorphometry BIC	The results using BIC values suggest that the Ca-P coating had no effect on improving bone apposition.
Poulos et al. 2011 [17]	Rabbit model (20)	40	2 and 4	Histomorphometry BIC	The porous titanium oxide implant coated with calcium phosphate behaved similarly to the porous titanium oxide control.
Quaranta et al. 2010 [18]	Rabbit model (12)	48	3, 4, and 8	Histomorphometry BIC	Ca-P coatings were osteoconductive and promoted early bone response.
Fügl et al. 2009 [19]	Non-human primate model (9)	25	80	Histomorphometry BIC	Ca-P coating of implants enhances osteoconductive properties in the initial phase.
Le Guehennec et al. 2008 [20]	Rabbit model (20)	40	2 and 8	Histomorphometry BIC	Higher BIC for the titanium implant coated with biomimetic Ca-P as compared with the grit-blasted implants. The osseointegration of Ca-P-Ti was similar to that observed for implants with etched surfaces.
Schliephake et al. 2006 [21]	Foxhound dog model (10)	10	4 and 12	Histomorphometry BIC	Coating an implant with Ca-P may have a beneficial effect on peri-implant bone regeneration and could improve BIC in the early stages of healing.
Caulier et al. 1997 [22]	Goat model (16)	64	16	Histomorphometry BIC	No final conclusion can be drawn due to the difference in surface roughness between the coated and noncoated implants.

Ca-P, calcium phosphate; BIC, bone-to-implant contact; Ti, titanium.

**Table 4:** Characteristics of implants.

Studies	Implant Dimensions, D(Ø) × L (mm)	Implant Shape	Ca-P Incorporation	Surface Coating
Koh et al. 2013 [15]	3.5 Ø × 8	Screw	Anodization	Mixed HA and CaO
Fontana et al. 2011 [16]	3.75 Ø × 7	Screw	Oxidation	Ca-P
Poulos et al. 2011 [17]	3.75 Ø × 7	Screw	Proprietary method (Nobel Biocare^®^)	Ca-P
Quaranta et al. 2010 [18]	4.5 Ø × 6	Screw	Ion beam-assisted deposition	Ca-P
Fügl et al. 2009 [19]	3 Ø × 10	Screw	Magnetron-sputtered	Ca-P
Le Guehennec et al. 2008 [20]	4.2 Ø × 6	Screw	Blasting	BCa-P
Schliephake et al. 2006 [21]	4 Ø × NR	Screw	Cathodic polarization	Ca-P
Caulier et al. 1997 [22]	3.75 Ø × 10	Screw	Plasma-spray	Ca-P

HA, hydroxyapatite; CaO, calcium oxide; Ca-P, calcium phosphate; BCa-P, bicalcium phosphate; NR, not reported.

**Table 5:** Meta-analysis of BIC according to random-effects model.

Study or Subgroup	Exp. Ca-P		Ti Sandblaster				Mean Difference		Year
	Mean	SD	Total	Mean	SD	Total	Weight	IV, Random, 95% CI	
Caulier et al.	67.4	27	64	26.5	16.2	64	12.2%	40.90 [33.19, 48.61]	1997
Schliephake et al.	45.2	9	10	31.5	10.8	10	12.0%	13.70 [4.99, 22.41]	2006
Le Guehennec et al.	47.3	3.9	40	68	3.9	40	12.9%	−20.70 [−22.41, −18.99]	2008
Fügl et al.	74.9	0.98	25	73.2	17	25	12.4%	1.70 [−4.97, 8.37]	2009
Quaranta et al.	43	3	48	31.5	2.4	48	13.0%	11.50 [10.41, 12.59]	2010
Fontana et al.	31.37	17.79	216	27.68	14.66	216	12.9%	3.69 [0.62, 6.76]	2011
Poulos et al.	73.5	4.2	40	79.4	2.8	40	13.0%	−5.90 [−7.46, −4.34]	2011
Koh et al.	53.7	10.9	12	53.6	15.8	12	11.6%	0.10 [−10.76, 10.96]	2013
**Total (95% CI)**			**455**			**455**	**100.0%**	**5.40 [−5.85, 16.65]**	

Herogeneity: Tau^2^ = 253.60; Chi^2^ = 1166.29, df = 7 (*P* < 0.00001): I^2^ = 99%. Test for overall effect: Z = 0.94 (*P* = 0.35). SD, standard deviation; CI, confidence interval.

## Data Availability

Not applicable.

## Abbreviations

BA	Bone area
BCa-P	Bicalcium phosphate
BD	Bone density
BIC	Bone-to-implant contact
Ca-P	Calcium phosphate
CAO	Calcium oxide
HA	Hydroxyapatite
MeSH	Medical Subject Headings
SLA	Sandblasted large grit acid etched

## Appendix A

**Table A1:** PRISMA Checklist.

Section/Topic	#	Checklist Item	Reported on Page #
**TITLE**			
Title	1	Identify the report as a systematic review, meta-analysis, or both.	1
**ABSTRACT**			
Structured summary	2	Provide a structured summary including, as applicable: background; objectives; data sources; study eligibility criteria, participants, and interventions; study appraisal and synthesis methods; results; limitations; conclusions and implications of key findings; systematic review registration number.	1
**INTRODUCTION**			
Rationale	3	Describe the rationale for the review in the context of what is already known.	2
**METHODS**			
Objectives	4	Provide an explicit statement of questions being addressed with reference to participants, interventions, comparisons, outcomes, and study design (PICOS).	2
Protocol and registration	5	Indicate if a review protocol exists, if and where it can be accessed (e.g., Web address), and, if available, provide registration information including registration number.	2
Eligibility criteria	6	Specify study characteristics (e.g., PICOS, length of follow-up) and report characteristics (e.g., years considered, language, publication status) used as criteria for eligibility, giving rationale.	2
Information sources	7	Describe all information sources (e.g., databases with dates of coverage, contact with study authors to identify additional studies) in the search and date last searched.	2
Search	8	Present full electronic search strategy for at least one database, including any limits used, such that it could be repeated.	2
Study selection	9	State the process for selecting studies (i.e., screening, eligibility, included in systematic review, and, if applicable, included in the meta-analysis).	8
Data collection process	10	Describe method of data extraction from reports (e.g., piloted forms, independently, in duplicate) and any processes for obtaining and confirming data from investigators.	3
Data items	11	List and define all variables for which data were sought (e.g., PICOS, funding sources) and any assumptions and simplifications made.	2
Risk of bias in individual studies	12	Describe methods used for assessing risk of bias of individual studies (including specification of whether this was done at the study or outcome level), and how this information is to be used in any data synthesis.	6
Summary measures	13	State the principal summary measures (e.g., risk ratio, difference in means).	3
Synthesis of results	14	Describe the methods of handling data and combining results of studies, if done, including measures of consistency (e.g., I^2^) for each meta-analysis.	7,8
